# Lupus Nephritis During the COVID-19 Pandemic: Challenges and Implications Before, During, and After

**DOI:** 10.3390/biomedicines13122984

**Published:** 2025-12-04

**Authors:** Michał Komorniczak, Piotr Stępniewski, Barbara Bułło-Piontecka, Katarzyna Aleksandra Lisowska, Alicja Dębska-Ślizień, Anna Wardowska

**Affiliations:** 1 Clinic and Department of Nephrology, Transplantology and Internal Diseases, Faculty of Medicine, Medical University of Gdańsk, 80-214 Gdańsk, Poland; bbullo@gumed.edu.pl (B.B.-P.); adeb@gumed.edu.pl (A.D.-Ś.); 2 Student Research Association at the Department of Nephrology, Transplantology, and Internal Diseases, Medical University of Gdańsk, 80-214 Gdańsk, Poland; piotrek9515@gmail.com; 3 Clinic and Department of Rheumatology, Clinical Immunology, Geriatrics and Internal Medicine, Faculty of Medicine, Medical University of Gdańsk, 80-210 Gdańsk, Poland; katarzyna.lisowska@gumed.edu.pl

**Keywords:** lupus nephritis, systemic lupus erythematosus, kidney transplantation, COVID-19, SARS-CoV-2, immunosuppression, renal outcomes

## Abstract

**Background:** Patients with systemic lupus erythematosus (SLE), particularly those with lupus nephritis (LN), are at increased risk of severe COVID-19 outcomes due to immune dysregulation and immunosuppressive therapy. Renal transplant recipients with prior LN (LN-RTX) combine chronic immunosuppression with residual autoimmune risk. This study aimed to evaluate renal function trajectories and disease activity in LN patients during the COVID-19 pandemic, comparing transplant recipients with conservatively managed patients (LN-CT), and to assess the potential effects of COVID-19 vaccination. **Methods:** A retrospective cohort of 111 biopsy-confirmed LN patients followed between 2019 and 2023 was analyzed at four time points: pre-pandemic (T0), first pandemic year (T1), second pandemic year (T2), and post-pandemic (T3). Changes in renal function, disease activity markers, and treatment patterns were compared between LN-CT (*n* = 100) and LN-RTX (*n* = 11) groups, with additional analysis by vaccination status. **Results:** Renal function declined significantly in LN-CT (median eGFR: from 76.5 to 66.5 mL/min/1.73 m^2^; *p* < 0.001) but remained stable in LN-RTX patients despite higher comorbidity rates. LN activity (proteinuria, erythrocyturia) and glucocorticoid/MMF use decreased over time. Vaccinated patients showed a statistically significant decline in eGFR (*p* = 0.00046), though causality remains uncertain. **Conclusions:** Renal outcomes during the pandemic differed by treatment modality, with LN-RTX patients maintaining stable function despite higher risk. Adjusted immunosuppression and vigilant monitoring may have supported these outcomes. Further prospective studies should clarify vaccine-related renal effects in LN populations.

## 1. Introduction

The COVID-19 pandemic had a profound impact on healthcare systems worldwide, presenting unique medical challenges. As of August 2023, there were approximately 770 million confirmed COVID-19 cases and around 6.9 million confirmed deaths [1]. This disease, caused by the novel severe acute respiratory syndrome coronavirus 2 (SARS-CoV-2), presents a range of symptoms [2,3]. Although SARS-CoV-2 primarily targets the respiratory system, its effects on individuals with pre-existing health conditions have been a significant focus of research and clinical concern. Among the most vulnerable groups are the elderly, the obese, and immunocompromised individuals, including renal transplant recipients (RTX) and patients suffering from autoimmune conditions such as systemic lupus erythematosus (SLE), particularly lupus nephritis (LN) [4,5,6,7,8,9,10,11].

SLE is a multisystem, chronic autoimmune disorder characterized by periods of remission and relapse. The pathogenesis of SLE involves dysregulation of innate and adaptive immune responses, leading to excessive production of autoantibodies, cytokine dysregulation, tissue inflammation, and organ damage. Central to this process is the presentation of autoantigens, which may result from impaired clearance of apoptotic cells. The presence of autoantigens triggers chronic immune activation and a feedback loop involving autoreactive B and T cells, complement activation, and pro-inflammatory cytokines [12,13,14,15]. LN, a common and severe renal complication of SLE, manifests clinically through proteinuria, hematuria, impaired renal function, edema, and hypertension [16]. The condition arises from dysregulated immune responses involving autoantibody production, complement activation, and cytokine disturbances, contributing to significant renal injury [13,17]. It may progress to chronic kidney disease or end-stage renal disease if inadequately managed [16,17]. Histologically, LN is divided into six classes based on renal biopsy findings, with immune-complex deposition and inflammatory infiltration playing central roles in its pathogenesis. Renal biopsy classification is essential for guiding therapy and predicting clinical outcomes in patients with LN [18,19].

Given their immunopathological similarities with COVID-19, including cytokine storms, complement activation, dysregulated interferon responses, and neutrophil extracellular traps (NETs), autoimmune diseases are expected to present more frequently and severely in the context of SARS-CoV-2 infection [20,21,22,23]. Multiple studies have documented such occurrences, reporting autoimmune manifestations, including Guillain–Barré syndrome [24,25,26,27,28], Kawasaki disease [29,30,31,32,33], SLE [34], hemolytic anemia [35,36,37], atypical hemolytic-uremic syndrome [38,39,40], and immune thrombocytopenic purpura [41,42,43].

For SLE, the emerging evidence indicates an increased risk of disease exacerbation and renal involvement during or after COVID-19. However, detailed data, particularly addressing LN among renal transplant recipients, remain limited and warrant further exploration [44,45,46,47]. Most studies published on SLE and COVID-19 describe large numbers of patients with SLE, with a relatively small number of COVID-19 confirmed cases [48]. Only a few reports of LN occurring after COVID-19 infection have been published [44,45,46]. Patients with SLE represent a group particularly susceptible to severe COVID-19 outcomes, mainly due to their inherent dysregulated immune responses and predisposition to cytokine dysregulation. Comprehensive data on LN among RTX patients with COVID-19 remain scarce, highlighting the need for further focused investigation [49,50,51].

RTX patients represent a particularly vulnerable group in the COVID-19 pandemic. Mortality among RTX patients infected with SARS-CoV-2 is considerably higher compared to the general population, especially during the early stages of the pandemic. Management strategies for RTX patients with COVID-19 depend on disease severity; mild cases generally require reduced doses of antimetabolites. At the same time, moderate to severe infections demand more substantial adjustments, including discontinuation of antimetabolites and dose reductions of calcineurin inhibitors or mammalian target of rapamycin (mTOR) inhibitors [52,53]. Glucocorticoids remain essential for managing severe COVID-19 presentations in RTX patients [54,55,56,57]. The clinical management of COVID-19 in renal transplant recipients requires careful management of immunosuppression to mitigate infection severity without jeopardizing graft survival. Optimizing immunosuppression and ensuring adequate vaccination remain pivotal strategies to improve clinical outcomes in this high-risk patient population. Given suboptimal immunogenicity of standard vaccination regimens, booster vaccinations, particularly heterologous regimens, have become essential for enhancing immune protection in RTX populations [58,59].

This retrospective, observational study aims to evaluate the impact of the COVID-19 pandemic and vaccination on renal outcomes in patients with biopsy-confirmed lupus nephritis (LN). We compare kidney transplant recipients (LN-RTX) with conservatively treated patients (LN-CT) across four pandemic phases (2019–2023), assessing longitudinal changes in renal function, immunosuppressive and supportive therapies, and access to clinical care. In addition, we investigate the relationship between COVID-19 vaccination status and kidney function in both groups, aiming to identify modifiable clinical and systemic factors that may impact long-term renal outcomes in this high-risk population.

## 2. Materials and Methods

### 2.1. Study Design

**Figure 1:**
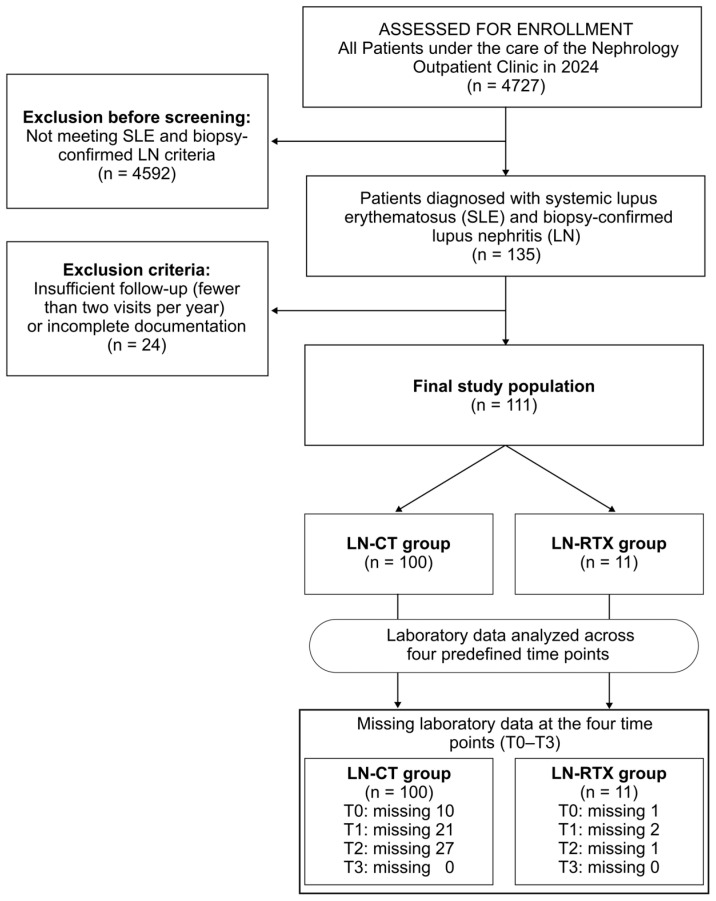
Flow diagram of cohort selection, exclusions, and data completeness across predefined time points (T0–T3). Among 4727 patients followed at the Nephrology Outpatient Clinic in 2024, 135 met diagnostic criteria for systemic lupus erythematosus (SLE) and had biopsy-confirmed lupus nephritis (LN). Patients with insufficient longitudinal follow-up (i.e., attending fewer than two nephrology visits per year since 2019) or incomplete documentation (*n* = 24) were excluded, resulting in a final study population of 111 individuals with complete clinical records across T0–T3. The cohort was stratified into two groups: patients with preserved native kidney function receiving conservative treatment (LN-CT, *n* = 100) and kidney transplant recipients with a history of LN (LN-RTX, *n* = 11). Laboratory data availability for each time point (T0–T3) is presented in the lower panel, illustrating patterns of missing data during follow-up.

Patients with LN enrolled in this study (*n* = 111) were selected from all individuals under the care of the Nephrology Outpatient Clinic (*n* = 4727) in 2024. Each patient met the diagnostic criteria for systemic lupus erythematosus applicable at the time and had biopsy-confirmed lupus nephritis. Only patients who attended at least two visits per year from 2019 onward were included. Among the LN patients, an LN-RTX group (*n* = 11) was distinguished (Figure 1).

The study was designed as a retrospective, longitudinal observational analysis to evaluate outcomes in patients with LN lupus nephritis during the COVID-19 pandemic. The analysis focused on clinical and laboratory findings at four time points: (i) T0, defined as the period before the COVID-19 pandemic (the year 2019); (ii) T1, the first year of the pandemic (2020); (iii) T2, the second year of the pandemic (2021); and (iv) T3, the period after the pandemic (2023).

Patients were evaluated for renal parameters, including serum creatinine and estimated glomerular filtration rate calculated using the CKD-EPI formula; inflammatory marker (C-reactive protein, CRP), and hematological parameters, including hemoglobin concentration, white blood cell count (WBC) with differentiation into neutrophils and lymphocytes, and platelet count. Features of hemolysis, such as lactate dehydrogenase levels, were also assessed.

The patient’s medical records were reviewed to assess prescribed and administered medications. Data on significant COVID-19 complications and vaccination status were also collected. Due to the use of immunosuppressive therapies, full vaccination was defined as completing the primary series, which included two doses of Comirnaty (Pfizer-BioNTech), Spikevax (Moderna), or Vaxzevria (AstraZeneca) vaccines, or one dose of Janssen (Johnson & Johnson), in addition to a booster dose [60,61]. Partial vaccination was defined as completing only the primary series.

### 2.2. Clinical Assessment of Lupus Nephritis

LN activity was classified as active if at least twice either proteinuria or erythrocyturia was detected. Proteinuria was defined as protein excretion of at least two plus on dipstick testing, 0.5 g per day or more in 24 h urine collection, or a urine protein-to-creatinine ratio (UPCR) of 500 milligrams of protein per gram of creatinine or more significant in a spot urine sample. Erythrocyturia was defined as the presence of at least five red blood cells per high-power field in the urine sediment, without any other identifiable cause apart from lupus nephritis.

Erythrocyturia was assessed semi-quantitatively using a modified ranking system based on the number of red blood cells (RBCs) observed per high-power field (HPF) in urine sediment, adapted from previously published scoring methodologies [62,63]. In this modified approach, ranks were assigned as follows: fewer than 3 RBCs/HPF scored 0 points; 4–10 RBCs/HPF scored 1 point; 11–15 RBCs/HPF scored 2 points; and more than 15 RBCs/HPF scored 3 points (maximum score). This standardized, quantitative evaluation of erythrocyturia provides a reliable basis for statistical analysis and enables accurate comparisons across study groups.

Renal function was further analyzed for clinically significant decline, defined as an increase in serum creatinine greater than 25%, or a reduction in eGFR exceeding 30%, or the initiation of renal replacement therapy (RRT), such as dialysis or kidney transplantation, between consecutive evaluation points.

### 2.3. Laboratory Measurements

Serum creatinine was measured by spectrophotometry, with reference ranges of 0.55–1.02 mg/dL for women and 0.73–1.18 mg/dL for men. C-reactive protein (CRP) levels were determined by immunoturbidimetric assay, with a reference range of 0.0–5.0 mg/L.

Urinalysis with sediment evaluation was performed using dry-chemistry test strips, and urine sediment was analyzed under a phase-contrast microscope at 400× magnification. The reference value for red blood cells in the urine sediment was up to 3 per HPF.

CBC was conducted on venous blood samples collected in ethylenediaminetetraacetic acid (EDTA) tubes. Hemoglobin concentration was measured spectrophotometrically, with reference ranges of 12.0–15.0 g/dL for women and 13.0–17.0 g/dL for men. Additional blood parameters, including WBC (reference range: 4.0–10.0 × 10^9^/L), neutrophil count (2.0–7.0 × 10^9^/L), and lymphocyte count (1.0–3.0 × 10^9^/L), were assessed using flow cytometry. Platelet counts (PLT) were evaluated using the hydrodynamic focusing method, with a reference range of 150–410 × 10^9^/L.

Proteinuria was measured by turbidimetric methods, either as 24 h urinary protein excretion or UPCR.

### 2.4. Statistical Analysis

Continuous variables were expressed as means ± standard deviation (SD) for normally distributed data or as medians with interquartile ranges (IQRs) for non-normally distributed data. Normality of distribution was assessed using the Shapiro–Wilk test. Categorical variables were summarized as absolute counts and percentages. Comparisons between two independent groups were performed using the Mann–Whitney U test for non-normally distributed variables. Paired comparisons of two measurements were analyzed using the Wilcoxon signed-rank test. For multiple comparisons within a group, the Friedman ANOVA was applied, followed by Dunn’s post hoc test when appropriate. Repeated-measures analysis of variance (ANOVA) was used for normally distributed repeated measures. Comparisons between categorical variables were conducted using Pearson’s Chi-square test or Fisher’s exact test when expected cell frequencies were <5.

To evaluate longitudinal renal function trajectories and assess whether COVID-19 vaccination independently influenced serum creatinine or eGFR over time, multivariable linear mixed-effects models were constructed. These models included a random intercept for each participant (ID) to account for intra-individual correlation across repeated measurements and incorporated time, vaccination status, and the time × vaccination interaction as primary predictors. Covariates included age, sex, hypertension, diabetes, non-glucocorticoid immunosuppression, daily glucocorticoid dose (prednisone-equivalent), and active nephritis. β coefficients and 95% confidence intervals (CIs) were reported.

The mixed-effects framework was chosen due to its robustness in handling incomplete and irregularly spaced longitudinal data under a missing-at-random assumption. Model assumptions were assessed through inspection of residuals and evaluation of fit indices. Missing data were handled using an available-case approach without imputation.

The sample size was informed by preliminary laboratory data from the same cohort, assuming a detectable within-subject difference with 80% power and a two-sided α of 0.05 using a paired-samples framework.

A two-sided *p*-value < 0.05 was considered statistically significant. In tables, only statistically significant *p*-values are reported; non-significant comparisons are marked as NS (non-significant). For highly significant results, *p*-values less than 0.001 were uniformly reported as *p* < 0.001. Selected non-significant *p*-values are reported in the text when deemed clinically or interpretatively relevant. Unless otherwise stated, data are presented as medians with interquartile ranges (25th–75th percentile). All statistical analyses were performed using Statistica version 13.3 (TIBCO Software Inc., Palo Alto, CA, USA) and R version 4.3.2 (R Foundation for Statistical Computing, Vienna, Austria) for the mixed-effects models.

## 3. Results

### 3.1. Characteristics of the Study Groups

A total of 111 patients were included in the study, comprising 91 women and 20 men, with a mean age of 46.5 years (±13.04). The median follow-up duration in the Nephrology Outpatient Clinic was 9 years (3–9 years). Among the participants, 11 patients (9.9%) had undergone kidney transplantation before the COVID-19 pandemic (LN-RTX group). The remaining 100 patients (90.1%) were treated conservatively for lupus nephritis (LN-CT group). The complete characteristics of both groups are presented in Table 1.

The groups differed significantly in the prevalence of diabetes, with 54.5% of the LN-RTX group and 8% of the LN-CT group (*p* = 0.0004). Moreover, significant differences were observed in the use of immunosuppressive therapy, with 100% of the LN-RTX group and 57% of the LN-CT group receiving it (*p* = 0.007). A particularly notable difference was observed in the proportion of patients receiving calcineurin inhibitors, with 100% of the LN-RTX group versus 13% of the LN-CT group (*p* < 0.001). There was a numerical difference in mycophenolate mofetil use between the groups, with 81.8% of LN-RTX and 48.0% of LN-CT patients receiving this treatment; however, this difference did not reach statistical significance (*p* = 0.0535).

### 3.2. Serum Creatinine, eGFR, and Clinically Significant Renal Function Decline

**Figure 2:**
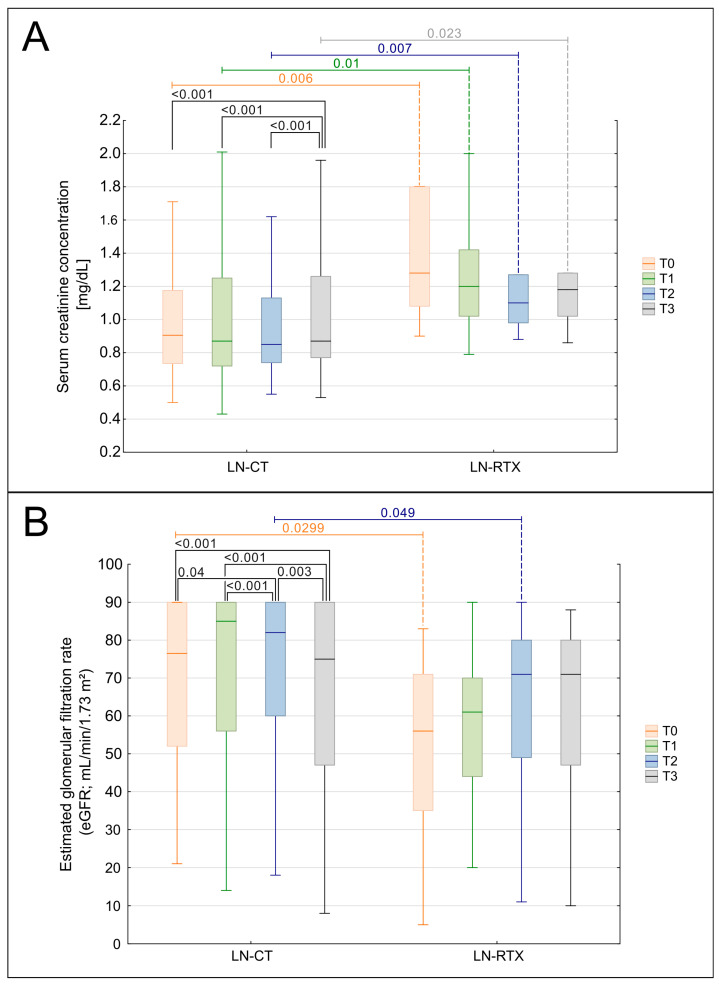
Comparison of kidney function over time in LN-CT and LN-RTX. Longitudinal analysis of renal function parameters presented as serum creatinine (**A**) and estimated glomerular filtration rate (eGFR) (**B**) in lupus nephritis patients managed conservatively (LN-CT, *n* = 100) versus renal transplant recipients (LN-RTX, *n* = 11). Laboratory findings were analyzed at four distinct time points: T0—the period preceding the COVID-19 pandemic (2019); T1—the first pandemic year (2020); T2—the second pandemic year (2021); and T3—the post-pandemic period (2023). Box-and-whisker plots illustrate the median, the interquartile range (IQR), and the minimum–maximum range. *p*-values shown on the plots denote between-group comparisons (LN-CT vs. LN-RTX) performed using the Mann–Whitney U test. Within-group comparisons across time points were assessed using the Kruskal–Wallis test with post hoc Wilcoxon tests. A two-tailed *p* < 0.05 was considered statistically significant.

A significant increase in serum creatinine concentration was observed throughout the observation period (T0-T3) in the entire cohort of patients (*p* = 0.0017; Figure 2A). However, the subgroup analysis revealed that this increase maintained its significance only in the LN-CT group (*p* = 0.00067), not in the LN-RTX patients. Interestingly, this parameter was significantly higher in the LN-RTX group at the beginning of the observation in time point T0, compared to the LN-CT group (*p* = 0.006), with median values of 1.185 mg/dL (IQR: 1.08–1.67) vs. 0.905 mg/dL (IQR: 0.735–1.175), respectively (Figure 2A). This intergroup difference remained statistically significant at T1 (*p* = 0.01), T2 (*p* = 0.007), and T3 (*p* = 0.023).

Longitudinal analysis demonstrated a significant decline in eGFR across the entire cohort (*p* = 0.0017, Figure 2B). Subgroup analysis revealed that this decline was significant in the LN-CT group (*p* = 0.00023) but not in LN-RTX patients (*p* = 0.447). The differences between the groups were also detected when particular time points were compared. At the baseline assessment, T0, the LN-RTX group had significantly lower eGFR values (median: 57.5 mL/min/1.73 m^2^; IQR: 37–71) compared to the LN-CT group (median: 75.5 mL/min/1.73 m^2^; IQR: 52–90) (*p* = 0.0299). A significant difference between groups was also observed at T2 (*p* = 0.049), while intergroup differences at T1 and T3 did not reach statistical significance.

A clinically significant decline in renal function, defined as a ≥25% increase in serum creatinine or a ≥30% reduction in eGFR between two consecutive time points, was observed in 25 patients during the study period. The highest incidence occurred between T0 and T1 (*n* = 12), followed by 6 events between T1 and T2, and 7 between T2 and T3. These events predominantly affected the LN-CT group, with only 1, 1, and 0 patients from the LN-RTX group affected in the respective intervals.

Significantly, only one patient in the LN-CT group progressed to end-stage renal disease requiring changing the form of renal replacement therapy to hemodialysis. At the same time, no dialysis was needed in the LN-RTX group. These findings align with the longitudinal analyses, reinforcing the relative stability of renal function in transplant recipients compared to conservatively treated patients.

### 3.3. Lupus Nephritis Activity

At baseline (T0), lupus nephritis activity was present in 54.3% of patients, with a higher prevalence in the LN-CT group (56%) compared to the LN-RTX group (36.4%). Over the observation period, the proportion of patients with active disease declined non significantly, reaching 48.9% at T1, 39.8% at T2, and then rebounding to 48.9% at T3.

Patients with active lupus nephritis at T0, regardless of the assigned group, experienced a significant deterioration in renal function. Serum creatinine increased from 1.02 to 1.30 mg/dL (*p* = 0.0049), while eGFR declined from 72.5 to 69.3 mL/min/1.73 m^2^ (*p* = 0.012). In contrast, these changes were not statistically significant in patients without active nephritis at the first time point.

A decreasing trend in the prevalence of active proteinuria was observed throughout the study. In the overall cohort, the proportion of patients with proteinuria declined from approximately 73% at T0 to 59% at T1, 46% at T2, and 41% at T3. This trend was predominantly observed in the LN-CT group, with a significant decrease in the percentage of affected patients (from 78.8% at T0 to 42.9% at T3, *p* = 0.00039; Kendall’s W = 0.098). In contrast, the LN-RTX group showed lower and more stable proteinuria levels, ranging from 45% at T0 to 22% at T3. No significant change over time was observed in this group.

A gradual reduction in erythrocyturia scores was also noted. In the overall cohort, the mean erythrocyturia score declined from 0.59 at T0 to 0.31 at T1, 0.13 at T2, and 0.43 at T3. This trend was most noticeable in the LN-CT group, where values dropped from 0.60 to 0.11 by T2, then partially rebounded to 0.43 at T3. The LN-RTX group showed lower erythrocyturia scores, ranging from 0.50 at T0 to 0.43 at T3, without a consistent trend.

These findings collectively indicate a reduction in lupus nephritis activity over time, particularly in LN-TC patients, as reflected by improvements in proteinuria and erythrocyturia. This decrease in urinary markers of disease activity may be linked to changes in immunosuppressive treatment strategies during the pandemic.

### 3.4. C-Reactive Protein

CRP levels did not show significant changes throughout the study in either treatment group (Figure S1). In the LN-CT group, median CRP values remained relatively stable, measured at 1.49 mg/L at T0, 1.85 mg/L at T1, 1.68 mg/L at T2, and 1.75 mg/L at T3. A similar pattern was observed in the LN-RTX group, with median values of 5.16 mg/L at T0, 8.2 mg/L at T1, 2.30 mg/L at T2, and 2.70 mg/L at T3.

Although baseline CRP levels were higher in the LN-RTX group, the intergroup comparison did not reach statistical significance. Moreover, no considerable alterations were observed between the groups at any subsequent time point.

These results indicate negligible fluctuations in inflammation over the study period, as reflected by CRP levels, in both treatment cohorts.

### 3.5. Peripheral Complete Blood Counts

A comprehensive assessment of peripheral blood counts, including hemoglobin concentration, leukocyte subpopulations (total WBC, neutrophils, lymphocytes), and PLT, was performed across all time points in both study groups. All variables were analyzed as non-normally distributed and are presented as medians with interquartile ranges (Table S1).

No statistically significant changes over time were observed in any of the evaluated parameters, either in the entire cohort or in subgroup analyses (LN-CT vs. LN-RTX). Specifically, hemoglobin levels remained stable throughout the observation period, with no significant within-group variation in either LN-CT or LN-RTX. Similarly, total white blood cell counts showed no significant longitudinal changes, and no differences were observed between groups at individual time points.

Neutrophil and lymphocyte counts also remained stable over time in both treatment regimens, with no indication of clinically relevant cytopenia or compensatory shifts. Platelet counts showed mild fluctuations, which were not statistically or clinically significant.

Collectively, these results indicate that CBC profiles remained largely unaffected during the study period, regardless of treatment modality.

### 3.6. Modifications to Treatment Recommendations

A consolidated overview of key longitudinal changes in renal parameters, lupus nephritis activity markers, and therapeutic patterns across T0–T3 is presented in Table S5.

#### 3.6.1. Use of Glucocorticoids

Glucocorticoids (GCs) were administered to the vast majority of patients, with no statistically significant difference in GCs use frequency between the LN-CT and LN-RTX groups at any time point. At baseline (T0), GCs were used by 95% of patients in the LN-CT group and by 100% in the LN-RTX group. These proportions remained stable during the observation period, with minor fluctuations in the LN-CT group (T1: 93%, T2: 94%, T3: 93%).

**Figure 3:**
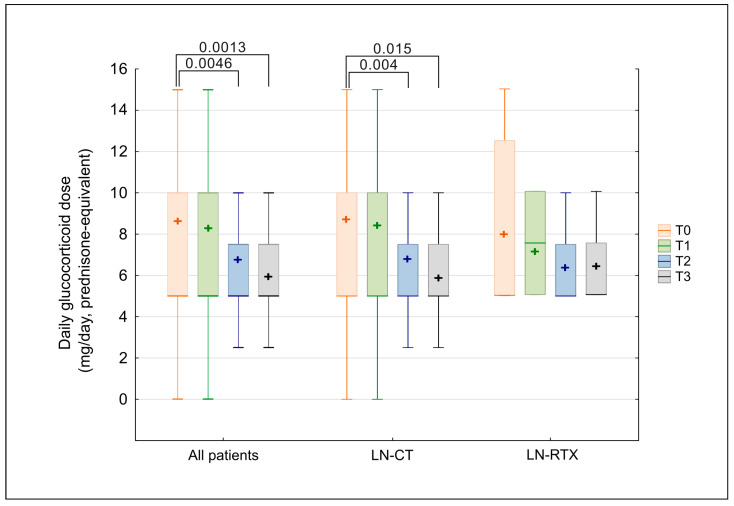
Comparison of glucocorticoid dose (prednisone-equivalent) over time in LN-CT and LN-RTX. Longitudinal assessment of daily glucocorticoid (GCS) dosage, expressed as prednisone-equivalent dose (mg/day), in patients with lupus nephritis who received GCS therapy (*n* = 71). Data are shown for the overall treated cohort (“All patients”) and for subgroups managed conservatively (LN-CT, *n* = 62) or after renal transplantation (LN-RTX, *n* = 9). Laboratory findings were analyzed at four time points: T0—the pre-pandemic period (2019); T1—the first pandemic year (2020); T2—the second pandemic year (2021); and T3—the post-pandemic period (2023). Box-and-whisker plots display medians and interquartile ranges (IQRs); whiskers indicate minimum and maximum values, and “+” symbols indicate mean values. Statistical comparisons between indicated time points were performed using the Kruskal–Wallis test with post hoc Wilcoxon tests; only significant *p*-values (*p* < 0.05) are presented. Between-group comparisons (LN-CT vs. LN-RTX) were performed using the Mann–Whitney U test; no statistically significant differences were detected at any time point.

Although the percentage of patients treated with GCS remained unchanged, the applied doses were significantly reduced over time. The longitudinal assessment of daily glucocorticoid dosage, expressed as a prednisone-equivalent dose (mg/day), showed a gradual decline over the study period (Figure 3). In the overall cohort, the median dose decreased significantly (*p* = 0.00053; Kendall’s W = 0.06057), with post hoc Wilcoxon testing confirming significant reductions beginning at observation time point T2 (T2 vs. T0: *p* = 0.0046; T3 vs. T0: *p* = 0.0013). No statistically significant differences in glucocorticoid doses were observed between the LN-CT and LN-RTX groups at any time point (Mann–Whitney U test, all *p* > 0.05).

The separate analysis of tested groups revealed a statistically significant reduction in the LN-CT group (*p* = 0.0053). At the same time, the pairwise comparisons confirmed lower doses at T2 (*p* = 0.004) and T3 (*p* = 0.015) relative to baseline. In the LN-RTX group, a similar trend was observed; however, it lacked statistical significance, likely due to the small sample size and wider variability.

#### 3.6.2. Immunosuppressive Therapy

Immunosuppressive agents were administered to 71 out of 110 patients (64.5%). While all patients in the LN-RTX group received such treatment (100%), its use was significantly lower in the LN-CT group (60%; *p* = 0.007). The overall prevalence of immunosuppressive therapy remained relatively stable over the study period (67.3% at T1, 67.3% at T2, and 65.7% at T3). In the LN-CT group, a slight increase in use was observed from 60.0% at baseline (T0) to 71.0% at T3. In contrast, the LN-RTX group consistently maintained a 100% use rate throughout the entire observation period. A significant difference between groups was observed only at baseline; no statistically significant differences were observed at subsequent time points (Fisher’s exact test).

**Figure 4:**
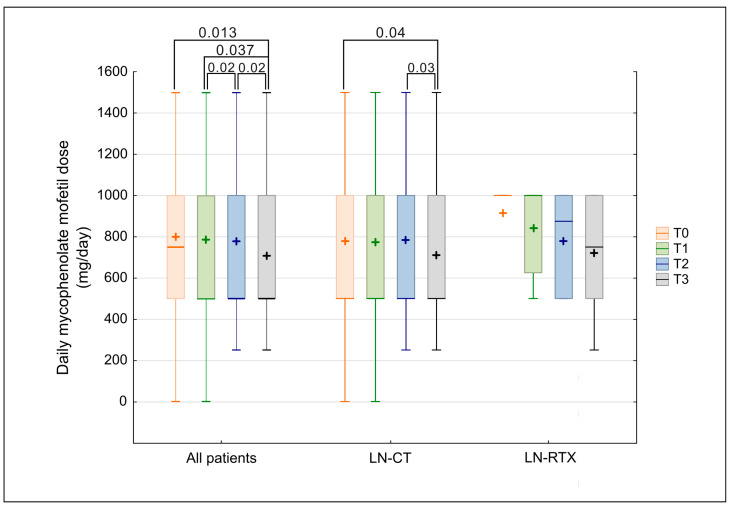
Comparison of mycophenolate mofetil dose over time in LN-CT and LN-RTX. Longitudinal comparison of daily mycophenolate mofetil dosage (mg/day) in the total cohort of lupus nephritis patients (“All patients”) and subgroups stratified according to treatment strategy: patients treated conservatively (LN-CT, *n* = 100) versus renal transplant recipients (LN-RTX, *n* = 11). Laboratory findings were analyzed at four distinct time points: T0—the period preceding the COVID-19 pandemic (2019); T1—the first pandemic year (2020); T2—the second pandemic year (2021); and T3—the post-pandemic period (2023). Box-and-whisker plots represent median values, interquartile ranges (IQRs), minimum and maximum values, and mean values indicated by “+” symbols. *p*-values represent statistically significant within-group differences between the indicated time points, as determined by a Kruskal–Wallis analysis followed by post hoc Wilcoxon tests. A two-tailed *p*-value < 0.05 was considered statistically significant. Between-group comparisons (LN-CT vs. LN-RTX) were assessed using the Mann–Whitney U test; no statistically significant differences were detected.

Mycophenolate mofetil (MMF) was the most frequently used immunosuppressive agent throughout the study period, with a higher prevalence in the LN-RTX group than in the LN-CT group (81.8% vs. 48.0%). Although the difference did not reach statistical significance (*p* = 0.0535), a numerical trend toward greater MMF use in the transplant group was observed. Among patients receiving MMF, daily doses did not differ between the two groups (LN-CT vs. LN-RTX) at any time point (Mann–Whitney U test, all *p* > 0.05). Despite this cross-sectional similarity, a significant longitudinal decline in MMF dosing was observed in the overall cohort between baseline (T0) and observation time point T3 (*p* = 0.0231), as shown in Figure 4. Subgroup analyses confirmed that this reduction was primarily driven by the LN-CT group, in which MMF doses decreased significantly between T0 and each subsequent time point. In contrast, MMF dosing remained stable in the LN-RTX group over time, with no statistically significant changes observed.

Other immunosuppressive agents, including azathioprine (AZA), cyclosporine A (CsA), and tacrolimus (TAC), did not exhibit significant temporal trends in use. However, TAC use was markedly more common in the LN-RTX group throughout the study period (approximately 72.7% vs. 2.5%; *p* < 0.001 across all time points). Cyclophosphamide (CYC), administered exclusively intravenously, was used only in the LN-CT group. In total, 11 of 100 LN-CT patients (11%) received CYC at least once during the observation period. Details on the timing and distribution of immunosuppressive therapies are provided in Table S2.

#### 3.6.3. Use of Antimalarial Drugs

At baseline (T0), antimalarial drugs were used by approximately one-third of all patients, with use more frequent in the LN-CT group (37.1%) than in the LN-RTX group (9.1%) (Table S2). Although not statistically significant, this observation may reflect differing baseline treatment strategies between conservatively treated and post-transplant patients.

During the observation period, a progressive increase in antimalarial drug use was observed across the entire cohort, with the proportion of patients receiving this treatment rising from 34.3% at T0 to 50.5% at T3. The difference between the initial and final time points was statistically significant (*p* = 0.0184), indicating a trend of increasing antimalarial therapy utilization throughout the pandemic.

#### 3.6.4. Use of Renin–Angiotensin–Aldosterone System Blockade

The use of angiotensin-converting enzyme inhibitors (ACE-Is) or angiotensin receptor blockers (ARBs) was observed in 55.0% of patients overall, with a higher rate in the LN-CT group than in the LN-RTX group (56.0% vs. 45.5%, Figure S2). Over the observation period, the proportion of patients receiving ACE-Is/ARBs therapy increased from 55.0% at baseline (T0) to 66.7% at T1, 68.5% at T2, and 64.9% at T3. Although this trend indicated a gradual increase over time in the total cohort, none of the pairwise comparisons between time points reached statistical significance.

When stratified by treatment group, a gradual increase in ACE-Is/ARBs use was observed only among patients who were conservatively treated. In contrast, usage patterns in the transplant group remained unchanged throughout the study period.

These findings suggest a non-significant tendency toward broader implementation of Renin–Angiotensin–Aldosterone System (RAAS) blockade during the COVID-19 pandemic period, possibly reflecting increased awareness of cardiovascular protection strategies.

#### 3.6.5. Use of Vitamin D

Vitamin D supplementation was assessed solely through physician-documented written recommendations; dosage and adherence data were not available.

**Figure 5:**
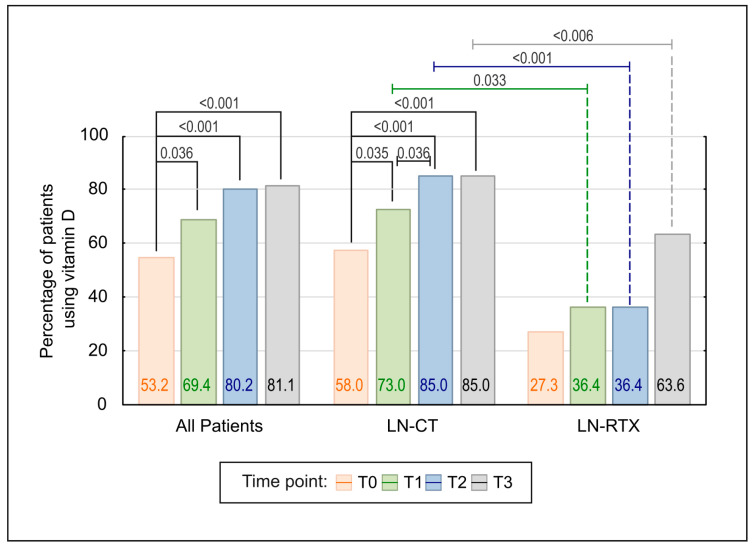
Comparison of Vitamin D use over time in LN-CT and LN-RTX patients. The bar graph illustrates the percentage of patients receiving vitamin D supplementation at four consecutive time points (T0–T3), both in the total cohort and stratified by treatment subgroup (LN-CT and LN-RTX). A statistically significant increase in vitamin D use was observed in the overall population (*p* < 0.001) and in the LN-CT subgroup (*p* < 0.001), with post hoc comparisons confirming significant differences between baseline (T0) and subsequent time points. A delayed but notable increase was also observed in the LN-RTX group, particularly between T0 and T3 (*p* < 0.001). Between-group comparisons revealed significantly lower supplementation rates in the LN-RTX group at multiple time points. Only statistically significant differences (*p* < 0.05) are shown. *p*-values above the bars reflect pairwise comparisons, calculated using the Wilcoxon signed-rank test or Fisher’s exact test, as appropriate.

A significant increase in vitamin D supplementation was observed across the study period (*p* < 0.001; Figure 5). The percentage of patients receiving vitamin D increased from 53,2% at baseline to 69.4%, 80.2%, and 81.1% at subsequent time points. Pairwise comparisons confirmed statistically significant increases from baseline to each subsequent time point (T1–T3), while no additional significant changes were observed beyond T1. Although the proportion of patients continued to increase after T1, the changes between later time points did not reach statistical significance, suggesting a plateau effect. This plateau effect may indicate a saturation point in vitamin D use, beyond which further increases may not significantly affect patient outcomes.

The stratified analysis confirmed a significant upward trend in vitamin D supplementation within the LN-CT group (*p* < 0.001), consistent increases observed from baseline through T3, including a significant stepwise rise between T1 and T2. No further increase in uptake was observed between T2 and T3, indicating a plateau. In contrast, the LN-RTX group showed no statistically significant change over time.

Between-group comparisons revealed consistently lower rates of vitamin D supplementation in the LN-RTX group, with statistically significant differences at T1 (*p* = 0.033), T2 (*p* < 0.001), and T3 (*p* < 0.006).

### 3.7. Vaccinations

In the LN-CT group, 72 out of 100 patients received at least one dose of a COVID-19 vaccine, with 50 completing the full vaccination schedule. In the LN-RTX group, 7 of 11 patients completed a primary vaccination series. Among fully vaccinated individuals in the LN-CT group, the majority (*n* = 36) received Comirnaty (Pfizer-BioNTech). Seven patients were initially vaccinated with Vaxzevria (AstraZeneca) but received a booster dose of Comirnaty; one patient received a primary series of Vaxzevria followed by a Spikevax (Moderna) booster, and two patients were vaccinated exclusively with Spikevax. Within the LN-CT subgroup of partially vaccinated patients (*n* = 22), 19 received Comirnaty, 2 received Vaxzevria, and 1 received the Janssen (Johnson & Johnson) vaccine. All vaccinated patients in the LN-RTX group received Comirnaty. Due to the small sample size of the LN-RTX group and uniform vaccination status, comparative analysis between treatment subgroups was not feasible.

**Figure 6:**
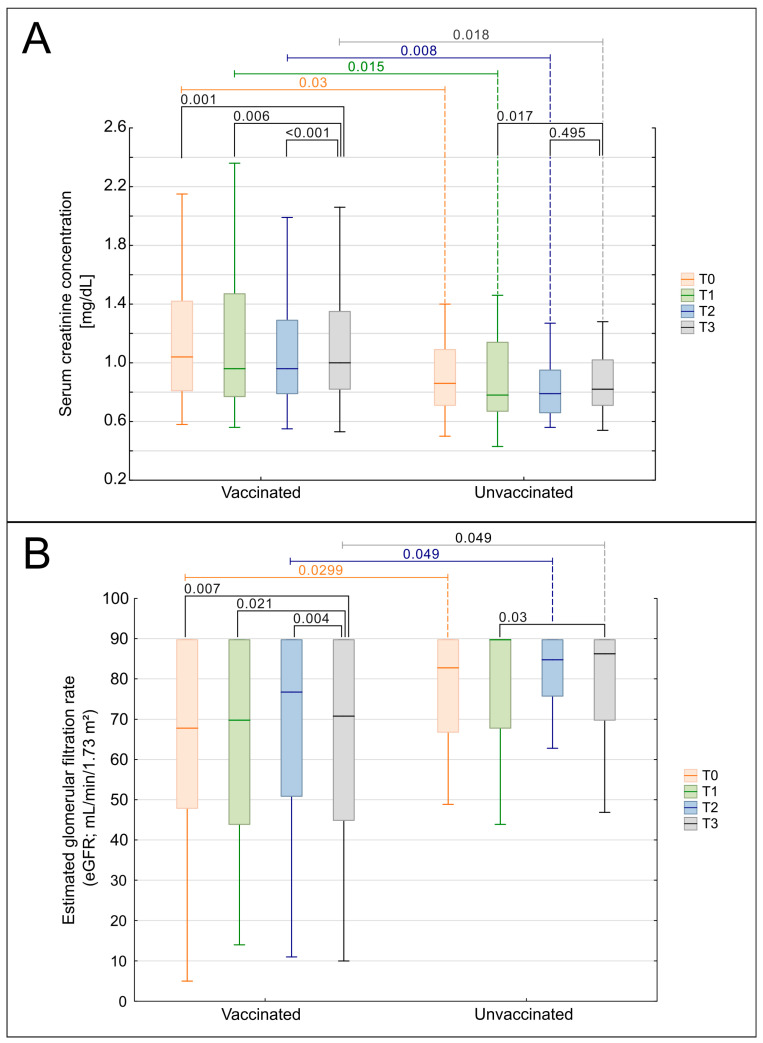
Comparison of renal function over time in vaccinated and unvaccinated patients. (**A**) Serum creatinine concentration (sCr, mg/dL) and (**B**) estimated glomerular filtration rate (eGFR, mL/min/1.73 m^2^; CKD-EPI) measured at four time points: T0—year preceding the COVID-19 pandemic (2019); T1—first pandemic year (2020); T2—second pandemic year (2021); and T3—post-pandemic period (2023). Patients were stratified into two groups: vaccinated (fully or partially vaccinated) and unvaccinated. Box-and-whisker plots display medians, 25th–75th percentiles, and non-outlier ranges. *p*-values shown on the plots indicate within-group pairwise comparisons between time points, calculated using the Wilcoxon signed-rank test. Between-group comparisons (vaccinated vs. unvaccinated), where performed, used the Mann–Whitney U test with continuity correction, but are not displayed. Only statistically significant results (*p* < 0.05) are shown.

The longitudinal analyses of eGFR and sCr were performed to explore potential associations between vaccination and renal function (Figure 6A,B). Patients were stratified by COVID-19 vaccination status into three groups: fully vaccinated (complete vaccination schedule), partially vaccinated (incomplete vaccination schedule), and unvaccinated. Kendall’s ANOVA demonstrated statistically significant deterioration in renal function parameters over the observation period within the vaccinated cohort. A progressive decline in eGFR (*p* = 0.00046, Figure 6B) and a concomitant increase in sCr (*p* = 0.00109, Figure 6A) were observed. Stratification by vaccination completeness revealed that the fully vaccinated subgroup showed statistically significant trends in both eGFR and sCr deterioration. Conversely, patients with incomplete vaccination showed significant changes only in eGFR (*p* = 0.01618), accompanied by stable sCr values. No statistically significant overall changes in eGFR or sCr were observed over time.

Although these unadjusted longitudinal trends could superficially suggest a potential association between COVID-19 vaccination status and renal function trajectories, such interpretations are inherently limited by baseline imbalances and the absence of adjustment for clinical confounders. To more rigorously evaluate these relationships, we performed multivariable linear mixed-effects analyses that incorporated age, sex, comorbidities, glucocorticoid dose, non-steroidal immunosuppression, and active nephritis. In these adjusted models, vaccination status did not independently influence the rate of change in serum creatinine or eGFR, and the time-by-vaccination interaction remained non-significant for both parameters. These findings suggest that the apparent differences observed in unadjusted analyses are likely due to underlying disease activity rather than vaccine effects. The detailed results of the adjusted models are presented below.

#### Adjusted Mixed-Effects Model Analysis

To determine whether COVID-19 vaccination independently influenced renal function trajectories, we constructed multivariable linear mixed-effects models for serum creatinine and eGFR. These models incorporated time, vaccination status, their interaction, and key clinical covariates, including age, sex, hypertension, diabetes, non-glucocorticoid immunosuppression, daily glucocorticoid dose, and active nephritis.

In these adjusted models, vaccination status was not associated with the rate of change in either serum creatinine or eGFR over time, and the Time × Vaccination interaction remained non-significant for both outcomes (sCr: *p* = 0.160; eGFR: *p* = 0.680). This indicates that COVID-19 vaccination did not modify renal function trajectories in this cohort, in contrast to the apparent patterns observed in unadjusted analyses.

In the overall population, eGFR declined modestly across the study period (β −1.67 mL/min/1.73 m^2^ per time point; *p* = 0.027), whereas changes in serum creatinine approached but did not reach significance (*p* = 0.060). Among all predictors included in the model, active nephritis emerged as the strongest independent determinant of renal impairment, being associated with higher serum creatinine (β +0.27 mg/dL; *p* = 0.005) and lower eGFR (β −3.95 mL/min/1.73 m^2^; *p* = 0.004). Other covariates, including age, hypertension, diabetes, and non-glucocorticoid immunosuppression, did not significantly influence longitudinal renal trends.

These findings demonstrate that the unadjusted differences observed between vaccinated and unvaccinated patients were attributable to baseline disease severity rather than vaccination effects, providing robust evidence that COVID-19 vaccination does not adversely affect renal function in patients with lupus nephritis. The full results of the adjusted mixed-effects models are presented in Table S4.

## 4. Discussion

In this longitudinal cohort of patients with biopsy-confirmed lupus nephritis, we observed distinct renal and clinical trajectories during the pandemic. Conservatively treated patients (LN-CT) exhibited a gradual decline in kidney function and reductions in markers of lupus nephritis activity. In contrast, renal parameters in the small group of transplant recipients (LN-RTX) appeared generally stable over time. Several therapeutic patterns also evolved during the observation period. Importantly, multivariable mixed-effects models demonstrated that COVID-19 vaccination was not independently associated with renal function decline, indicating that unadjusted differences reflected underlying disease activity rather than vaccine-related effects. These findings contribute to a more nuanced understanding of renal vulnerability in LN during the pandemic and underscore the importance of disease-activity-guided monitoring.

### 4.1. Role of Immunosuppression

The descriptive stability of renal parameters in the LN-RTX subgroup should be interpreted within the clinical context of post-transplant care, where patients typically receive structured follow-up and consistent immunosuppressive regimens. Immunosuppressive therapies are known to modulate systemic inflammation, and prior literature suggests that such regimens may blunt excessive inflammatory responses during viral infections, including COVID-19 [64,65,66,67]. However, our study was not designed to evaluate mechanistic pathways, and no immunological markers were assessed that would allow direct inference regarding cytokine-mediated injury or its modulation. Thus, while existing evidence supports the general concept that immunosuppression can influence inflammatory dynamics, the patterns observed in LN-RTX here should be viewed as descriptive and not as evidence of a distinct immunopathological response.

### 4.2. Clinical Surveillance and Quality of Care

Interestingly, the LN-RTX group exhibited a markedly higher prevalence of diabetes mellitus (54.5% vs. 8%), a well-established risk factor for adverse renal outcomes. Despite this unfavorable baseline characteristic, renal function in this subgroup appeared generally stable throughout the observation period. However, this pattern should be interpreted cautiously due to the small sample size and the selected nature of our cohort. This finding likely reflects the fact that LN-RTX patients receive more intensive and continuously maintained immunosuppression, which is generally effective in suppressing lupus nephritis activity and may contribute to greater clinical stability during routine follow-up. Moreover, kidney transplant recipients are typically subject to rigorous nephrological follow-up, including standardized monitoring of renal parameters, immunosuppressive drug levels, and infection risk, which may facilitate early detection of emerging abnormalities. This heightened surveillance may have facilitated early identification and management of clinical changes. Both subgroups met the requirement of at least two annual nephrology visits, indicating a selected cohort that remained engaged in care during the pandemic. Missing laboratory data were more common in LN-CT, consistent with greater variability rather than true differences in disease stability. In contrast, patients in the LN-CT group may have experienced greater variability in access to in-person assessments during the pandemic, potentially affecting the consistency of monitoring and the timeliness of therapeutic adjustments. These observations underscore the importance of structured clinical oversight and prompt therapeutic adjustment in LN care, while highlighting that differences between LN-CT and LN-RTX in this study may also reflect selection and survivor biases rather than intrinsic biological differences.

### 4.3. Pathophysiological Context: Cytokine Storm, Autoimmunity, and NETs

Data presented in this study align with the existing literature on the renal impact of COVID-19 and its management in autoimmune populations. Previous studies have highlighted the dual risks posed by SARS-CoV-2 infection: direct viral effects on renal tissue and systemic inflammatory damage mediated by cytokine storm [68,69]. Additionally, the similarities between the immunopathogenesis of SLE and severe COVID-19, including dysregulated interferon pathways and complement activation, provide a compelling rationale for studying these conditions in parallel [21]. Moreover, the role of NETs in exacerbating inflammation and immunothrombosis in both conditions underscores overlapping pathophysiological mechanisms [70]. Notably, Polish data reported by Dr. Malinowska et al. revealed stable kidney graft function among RTX patients post-COVID-19, with creatinine and eGFR values showing minimal variation before and after infection [71]. Furthermore, the lack of studies analyzing kidney transplant cohorts with secondary glomerulonephritis as distinct subgroups underscores the unique value of this investigation, which provides targeted insights into this population, often overlooked in broader transplant research.

The cytokine storm phenomenon, characterized by excessive production of pro-inflammatory cytokines, is a key driver of severe COVID-19 and has notable parallels in SLE. In both conditions, elevated levels of cytokines such as interleukin 6 (IL-6), tumor necrosis factor α (TNF-α), and type I interferons contribute to systemic inflammation, tissue damage, and thrombotic complications [64]. Recent data highlight how SARS-CoV-2 infection exacerbates immune dysregulation in LN patients. For instance, Lisowska et al. observed that LN patients with prior SARS-CoV-2 infection exhibited increased regulatory T cells and classical monocytes, along with elevated serum IL-6 levels, compared to uninfected LN patients or healthy controls [72]. These findings emphasize the compounded immunological burden faced by LN patients during the pandemic and underscore the importance of tailored therapeutic approaches to mitigate cytokine-mediated damage. Additionally, the observed changes in the B-cell and monocyte compartments in post-COVID-19 LN patients highlight potential biomarkers for disease monitoring and therapeutic intervention targets. Understanding these shifts could refine management strategies for SLE patients navigating the dual challenges of lupus nephritis and COVID-19.

### 4.4. COVID-19 Vaccination and Renal Function

Interpreting vaccination-related renal outcomes in lupus nephritis requires careful consideration of baseline disease characteristics. In unadjusted analyses, vaccinated patients—particularly within the LN-CT group—appeared to have higher serum creatinine and lower eGFR at selected time points; however, these differences reflected underlying disease severity. After multivariable adjustment for age, comorbidities, daily glucocorticoid dose, non-glucocorticoid immunosuppression, and active nephritis, vaccination status was not independently associated with renal function decline. The time-by-vaccination interaction remained non-significant for both serum creatinine and eGFR. A sensitivity analysis excluding the small LN-RTX subgroup yielded qualitatively identical results, supporting the robustness of these findings.

Although vaccine-induced immune activation has been linked to rare cases of glomerulonephritis or lupus nephritis flares [73], no such relapses or severe post-vaccination complications were observed in our cohort. All adverse reactions were mild and transient, consisting mainly of nonspecific systemic symptoms such as low-grade fever or fatigue. These findings should therefore be interpreted within the broader context of the well-established benefits of COVID-19 vaccination. Vaccination remains critical for reducing severe disease, hospitalization, and mortality in this high-risk population [74].

Our results indicate that renal monitoring in lupus nephritis should be guided primarily by underlying disease activity rather than vaccination status. Further research is warranted to clarify how immunologic activity, therapeutic burden, and SARS-CoV-2 exposure collectively shape renal trajectories; however, our findings do not support a direct vaccine-related mechanism of renal decline.

Future studies with larger, multi-center cohorts and comprehensive assessment of both natural SARS-CoV-2 infection history and vaccination-related immune responses are warranted to clarify these associations.

### 4.5. Therapeutic Evolution During the Pandemic

The observed decrease in lupus nephritis activity during the pandemic may reflect evolving treatment patterns and changes in clinical management. For example, the LN-RTX group had a higher rate of calcineurin inhibitor (CNI) use (100% vs. 13%), a finding consistent with post-transplant immunosuppression protocols. Although CNIs can reduce lupus nephritis activity and exert broader anti-inflammatory effects, these mechanisms cannot be evaluated within our dataset. However, pandemic-related disruptions in healthcare access may also have influenced the documentation of disease activity. Future studies should assess whether these trends persist and represent true clinical improvements rather than documentation effects.

In our cohort, glucocorticoid use remained consistently high, while daily doses progressively declined over the study period, with statistically significant reductions observed predominantly in the LN-CT group. These findings highlight a cautious yet systematic tapering of glucocorticoids throughout the pandemic, consistent with evolving treatment guidelines and improved disease control, while maintaining high treatment adherence and avoiding flares (preferably aiming for the lowest effective dose and shortest feasible duration, in line with expert guidance) [75,76].

The COVID-19 pandemic significantly impacted clinical decision-making and treatment recommendations, which evolved dynamically as new evidence emerged. Early in the pandemic, reports of potential benefits for specific therapies, such as vitamin D supplementation and antimalarial drugs, influenced prescribing practices. Although initially considered promising, some of these treatments were later shown to lack robust clinical support, based on emerging data. As the pandemic progressed, some early therapeutic optimism proved premature, while concerns about the adverse effects of specific treatments lacked clinical trial support. This evolving knowledge has shaped our understanding of the pros and cons of various interventions, ultimately leading to refined treatment protocols for patients, including those with lupus nephritis and other chronic conditions.

### 4.6. Vitamin D Recommendations During the Pandemic

The increased use of vitamin D supplementation during the observation period likely reflects growing clinical interest in its potential relevance to COVID-19 outcomes, particularly among patients with autoimmune disease. Emerging evidence in SLE populations suggested that adequate vitamin D status might be associated with more favorable post-infection courses [77], which may have contributed to more frequent written recommendations in clinical practice. Conversely, vitamin D deficiency has been linked to more severe COVID-19, including higher risks of respiratory complications and mortality [78], while meta-analytic data suggest that supplementation may be associated with improved clinical outcomes [79]. Higher serum vitamin D levels have been associated with less severe pulmonary involvement in COVID-19, whereas deficiency has been linked to increased mortality risk [80]. The observed increase in vitamin D supplementation among LN patients during this study reflects a higher frequency of documented, written recommendations, underscoring the growing recognition of vitamin D in clinical practice during the pandemic. This trend likely mirrors increased clinician awareness of vitamin D as a potentially modifiable factor discussed in early pandemic literature, particularly for patients with autoimmune dysregulation and renal involvement. Importantly, our dataset lacked information on vitamin D dosing or adherence, preventing dose–response or exposure-effect analyses. As a result, the observed trends reflect prescribing behavior rather than actual vitamin D intake or biochemical sufficiency.

In our cohort, the increased frequency of vitamin D recommendations did not correspond to discernible differences in renal outcomes. As serum 25-hydroxyvitamin D levels, dosing information, and adherence data were unavailable, these observations reflect prescribing patterns rather than biological exposure or measurable clinical effects.

### 4.7. Antimalarials During the Pandemic

Antimalarial drugs, particularly hydroxychloroquine (HCQ), are a cornerstone of standard therapy in systemic lupus erythematosus and lupus nephritis. Early in the COVID-19 pandemic, these agents gained additional attention after in vitro studies suggested potential antiviral activity against SARS-CoV-2 [81], fueling substantial clinical optimism despite limited evidence. Subsequent randomized trials and meta-analyses did not demonstrate meaningful treatment or prevention benefits for HCQ [82], while the combination of HCQ and azithromycin was associated with increased mortality due to cardiac toxicity [83].

In our study, we examined whether this early enthusiasm—and the parallel emergence of safety concerns—was reflected in the prescribing patterns documented in routine clinical practice, as early recommendations for HCQ use in COVID-19 appeared to influence prescribing behavior during the initial phase of the pandemic. The increased use of antimalarials observed in our cohort likely reflects both their established role in the management of lupus nephritis and clinicians’ responses to early reports of potential antiviral activity. However, contemporary evidence does not support HCQ or related agents for COVID-19 treatment due to a lack of efficacy and possible adverse effects, including the increased mortality reported with HCQ–azithromycin combination therapy [82,84]. This pattern illustrates how rapidly evolving evidence shaped clinical decision-making during the pandemic.

### 4.8. RAAS Inhibitors During the Pandemic

RAAS inhibitors, particularly ACE inhibitors and ARBs, gained considerable attention early in the COVID-19 pandemic because of the theoretical concern that RAAS blockade might increase susceptibility to SARS-CoV-2 through ACE2 upregulation [85]. However, large, randomized trials and population-based registries consistently demonstrated no increase in infection risk or severe outcomes among users of these agents [86,87]. Although additional immunomodulatory and endothelial-protective effects of RAAS inhibition have been discussed in the literature [88,89], these mechanisms were not assessed in our study, and their clinical relevance during the pandemic remains uncertain.

The differential use of RAAS inhibitors observed in our cohort reflects established clinical practice patterns rather than COVID-related changes in prescribing behavior. A substantially higher proportion of LN-CT patients received ACE-Is or ARBs at all time points. In contrast, use was lower in the LN-RTX group, a pattern consistent with transplant-specific caution regarding RAAS blockade, given concerns about renal perfusion and the risk of hemodynamic compromise in solitary graft kidneys [90,91]. In our analysis, RAAS use was not associated with differences in renal outcomes, and no signal of harm was observed. These findings suggest that continued use of ACE-Is and ARBs during the pandemic aligned with standard nephroprotective practice in lupus nephritis and was not adversely affected by earlier theoretical concerns related to SARS-CoV-2.

### 4.9. Healthcare Delivery and Clinical Oversight

In addition to immunological and pharmacological factors, differences in healthcare delivery during the pandemic may have influenced the consistency and completeness of clinical monitoring in the two groups. During the COVID-19 pandemic, access to in-person care was substantially reduced for most chronically ill patients, including those with lupus nephritis. However, kidney transplant recipients remained under structured post-transplant surveillance, which generally ensured more predictable access to in-person monitoring. This continuity enabled consistent monitoring of renal function and immunosuppressive drug levels, facilitating early detection of complications. In contrast, the LN-CT group demonstrated greater variability in the timing and completeness of laboratory assessments, reflecting broader disruptions in routine outpatient care during the pandemic.

These differences in clinical oversight may have contributed to the observed stability in renal function in the LN-RTX group, despite their higher baseline comorbidity burden. These observations highlight how disruptions in routine healthcare delivery can shape the consistency of clinical monitoring in high-risk autoimmune nephrology populations. Our findings indicate that differences in the structure and predictability of clinical interactions may help explain the variation in data completeness and monitoring patterns observed between the two groups.

### 4.10. Study Limitations

This study has several limitations that should be acknowledged. First, the lack of histopathological stratification of lupus nephritis limits our ability to perform a nuanced analysis of disease progression and treatment responses. Membranous LN, diffuse proliferative LN, and tubulointerstitial inflammation represent distinct entities with varying prognostic implications [92,93].

Second, the study cohort’s racial and ethnic homogeneity (entirely Caucasian) limits the generalizability of findings, as severe forms of SLE and LN are more prevalent in African American and Hispanic populations [94].

Third, the small number of LN-RTX patients limits the statistical power of subgroup analyses, a constraint further compounded by the single-center design, underscoring the need for larger, multicenter studies. Moreover, the retrospective design of this study, together with the absence of post-vaccination immune response data and the lack of systematic information on the timing, history, and severity of natural SARS-CoV-2 infections, limits our ability to fully characterize the interplay between vaccination, infection exposure, and renal outcomes.

Finally, although no severe post-vaccination complications were documented, the absence of systematic or quantitative reporting of mild and self-reported adverse events limits the granularity of our safety assessments. It may have led to underreporting of minor reactions. Moreover, because our cohort inherently represents a survivor population—excluding individuals who died or were lost to follow-up during the pandemic—a degree of survivorship bias is unavoidable, which may limit the generalizability of our findings and underestimates the broader spectrum of COVID-19-related outcomes in lupus nephritis.

## 5. Conclusions

This longitudinal single-center study documents changes in renal parameters and other clinical indicators relevant to lupus nephritis during the COVID-19 pandemic. Across the study period, we observed a modest decline in eGFR alongside generally stable measures of proteinuria, consistent with the longitudinal laboratory patterns identified in our analyses. Although unadjusted analyses suggested modest differences in renal trajectories by vaccination status, these patterns were not significant in multivariable mixed-effects models.

The results of this study highlight the need for future investigations into the interplay between lupus nephritis, COVID-19, and immunosuppressive therapy. Given the racial and ethnic homogeneity of our cohort, longitudinal studies in more diverse populations will be essential for identifying long-term trends and disparities in outcomes. Incorporating histopathological classifications and assessing post-vaccination immunologic responses may further clarify the interplay between SARS-CoV-2 exposure, vaccination, and lupus nephritis-related immune dysregulation. Another potential avenue for future research is the role of newer therapeutic agents—such as type I interferon inhibitors—in shaping immune responses and clinical trajectories in patients with lupus nephritis during and beyond the COVID-19 era.

Despite the challenges posed by the COVID-19 pandemic, our findings illustrate how patients with lupus nephritis—particularly transplant recipients—continued to receive structured care that shaped patterns of clinical monitoring during this period. Our findings highlight the importance of individualized treatment strategies that balance immunosuppression and disease stability while minimizing the risk of infection. These findings align with broader evidence supporting COVID-19 vaccination as a key protective measure for immunosuppressed patients, and no vaccine-related renal relapses or severe adverse events were observed in our cohort. Differences in clinical context and immunosuppressive regimens between LN-RTX and LN-CT patients highlight the need for further research to clarify how treatment intensity and patterns of care shape longitudinal monitoring in lupus nephritis. Interpretation of subgroup trends should take into account differences in data completeness between LN-CT and LN-RTX patients, as transplant recipients had more consistent laboratory monitoring throughout the pandemic. These distinctions reflect patterns of care rather than differences in renal outcomes and highlight the need for improved methodological approaches in future longitudinal studies. Together, these findings help inform a more nuanced approach to the care of patients with lupus nephritis during periods of evolving clinical and public health uncertainty.

## Figures and Tables

**Table 1:** Characteristics of the study groups.

Parameter	LN-CT (*n* = 100)	LN-RTX (*n* = 11)	*p*-Value
Female/Male ratio	82 (82%)/18 (18%)	9 (82%)/2 (18%)	NS
Age (years)	47.24 ± 13.4	39.6 ± 3.45	NS
Percentage of active nephropathy	57 (57%)	3 (27%)	NS
**Comorbidities**			
Arterial hypertension	79 (79%)	9 (81.8%)	NS
Diabetes mellitus	8 (8%)	6 (54.5%)	<0.001
Antiphospholipid syndrome	30 (30%)	4 (36.4%)	NS
Osteoporosis	31 (31%)	3 (27.3%)	NS
**Treatment**			
Glucocorticoids (GCS)	95 (95%)	11 (100%)	NS
Daily GCS dose, prednisone-equivalent (mg)	5 (5–10)	5 (5–13)	NS
Immunosuppressive therapy	60 (60%)	11 (100%)	0.007
-Cyclophosphamide (intravenous)	3 (3%)	0 (0%)	NS
-Calcineurin inhibitors	13 (13%)	11 (100%)	<0.001
● Cyclosporine	11 (11%)	3 (27.3%)	0.007
● Tacrolimus	2 (2%)	8 (72.7%)	<0.001
-Antiproliferative drugs:	49 (49%)	9 (81.8%)	NS
● Mycophenolate mofetil (MMF)	48 (48%)	9 (81.8%)	NS
● Daily MMF dose (mg/day)	500 (500–1000)	1000 (750–1000)	NS
● Azathioprine	1 (1%)	0 (0%)	NS
Antimalarial drugs	38 (38%)	1 (9.1%)	NS
ACE-Is/ARBs	59 (59%)	5 (45.5%)	NS
Statins	20 (20%)	3 (27.3%)	NS
Vitamin D supplements	56 (56%)	3 (27.3%)	NS
**Laboratory parameters**			
Serum creatinine (mg/dL)	0.905 (0.735–1.175)	1.28 (1.08–1.8)	0.006
Estimated glomerular filtration rate (eGFR, mL/min/1.73 m^2^)	76.5 (52–90)	56 (35–71)	0.0299
Hemoglobin (g/dL)	13.1 (11.7–14.35)	12.6 (11.2–13.2)	NS
White blood cell count (WBC, ×10^9^/L)	6.275 (4.975–8.6)	8.57 (6.54–9.87)	NS
-Neutrophil count (×10^9^/L)	3.7 (2.71–5.57)	5.17 (4.11–7.12)	NS
-Lymphocyte count (×10^9^/L)	1.535 (1.07–2.185)	1.7 (1.14–1.74)	NS
Platelet count (PLT, ×10^9^/L)	236 (197–273)	209 (187–286)	NS
C-reactive protein (CRP, mg/L)	1.485 (0.7–4.61)	1.28 (1.08–1.8)	0.0382
Lactate dehydrogenase (LDH, U/L)	195 (176–240.5)	197 (63–228)	NS

Data are presented as counts and percentages for categorical variables. Age is reported as mean ± standard deviation. All other continuous variables, due to non-normal distribution, are presented as medians with interquartile ranges (IQRs). Between-group comparisons were performed using Fisher’s exact test for categorical variables and the Mann–Whitney U test for continuous variables. A two-sided *p*-value < 0.05 was considered statistically significant. Only statistically significant differences are reported with corresponding *p*-values; non-significant comparisons are marked as NS (non-significant). Estimated glomerular filtration rate (eGFR) was calculated using the CKD-EPI formula. Abbreviations: ACE-Is—angiotensin-converting enzyme inhibitors; ARBs—angiotensin receptor blockers.

## Data Availability

The data presented in this study are available on reasonable request from the corresponding authors. The data are not publicly available due to privacy and ethical restrictions related to patient confidentiality.

## Supplementary Materials

The following supporting information can be downloaded at: https://www.mdpi.com/article/10.3390/biomedicines13122984/s1; Figure S1. Longitudinal changes in C-reactive protein levels in lupus nephritis patients during the COVID-19 pandemic; Figure S2. Trends in ACE-Is/ARBs use over the COVID-19 pandemic in patients with lupus nephritis; Figure S3. Comparison of renal function over time in fully and partially vaccinated patients; Table S1. Peripheral blood parameters over time (T0–T3) in LN-CT and LN-RTX groups; Table S2. Immunosuppressive therapy over time (T0–T3) in LN-CT and LN-RTX groups; Table S3. Lupus nephritis activity and renal parameters over time (T0–T3); Table S4. Adjusted linear mixed-effects models for changes in serum creatinine and eGFR (T0–T3); Table S5. Summary of major longitudinal changes in clinical parameters and treatments (T0–T3).

## Abbreviations

The following abbreviations are used in this manuscript:

ACE-Is	Angiotensin-Converting Enzyme Inhibitors
ARBs	Angiotensin Receptor Blockers
AZA	Azathioprine
CBC	Complete Blood Counts
CKD-EPI	Chronic Kidney Disease Epidemiology Collaboration
COVID-19	Coronavirus Disease 2019
CRP	C-Reactive Protein
CsA	Cyclosporine A
eGFR	Estimated Glomerular Filtration Rate
GCS	Glucocorticoids
HCQ	Hydroxychloroquine
HPF	High Power Field
IFN	Interferon
IL	Interleukin
IQR	Interquartile Range
LDH	Lactate Dehydrogenase
LN	Lupus Nephritis
LN-CT	Lupus Nephritis Conservatively Treated
LN-RTX	Lupus Nephritis in Renal Transplant Recipients
MMF	Mycophenolate Mofetil
mTOR	Mammalian Target of Rapamycin
NETs	Neutrophil Extracellular Traps
NS	Not Significant
PLT	Platelet Counts
RAAS	Renin–Angiotensin–Aldosterone System
RRT	Renal Replacement Therapy
RTX	Renal Transplant Recipients
SARS-CoV-2	Severe Acute Respiratory Syndrome Coronavirus 2
SLE	Systemic Lupus Erythematosus
sCr	Serum Creatinine
SD	Standard Deviation
TAC	Tacrolimus
T0–T3	Time Points (T0: pre-pandemic; T1: first pandemic year; T2: second pandemic year; T3: post-pandemic)
UPCR	Urine Protein-to-Creatinine Ratio
WBC	White Blood Cell Count
