# Supplementary material for: Lupus Nephritis During the COVID-19 Pandemic: Challenges and Implications Before, During, and After
Source: Biomedicines. 2025 Dec 4;13(12):2984. doi: 10.3390/biomedicines13122984 (PMC12731086; doi:10.3390/biomedicines13122984)
Supplement: Supplementary file 1 [file biomedicines-13-02984-s001.zip › biomedicines-3952952-supplementary.pdf]

biomedicines

MDPI

Supplementary material

Lupus Nephritis During the COVID-19 Pandemic: Challenges and Implications Before, During, and After

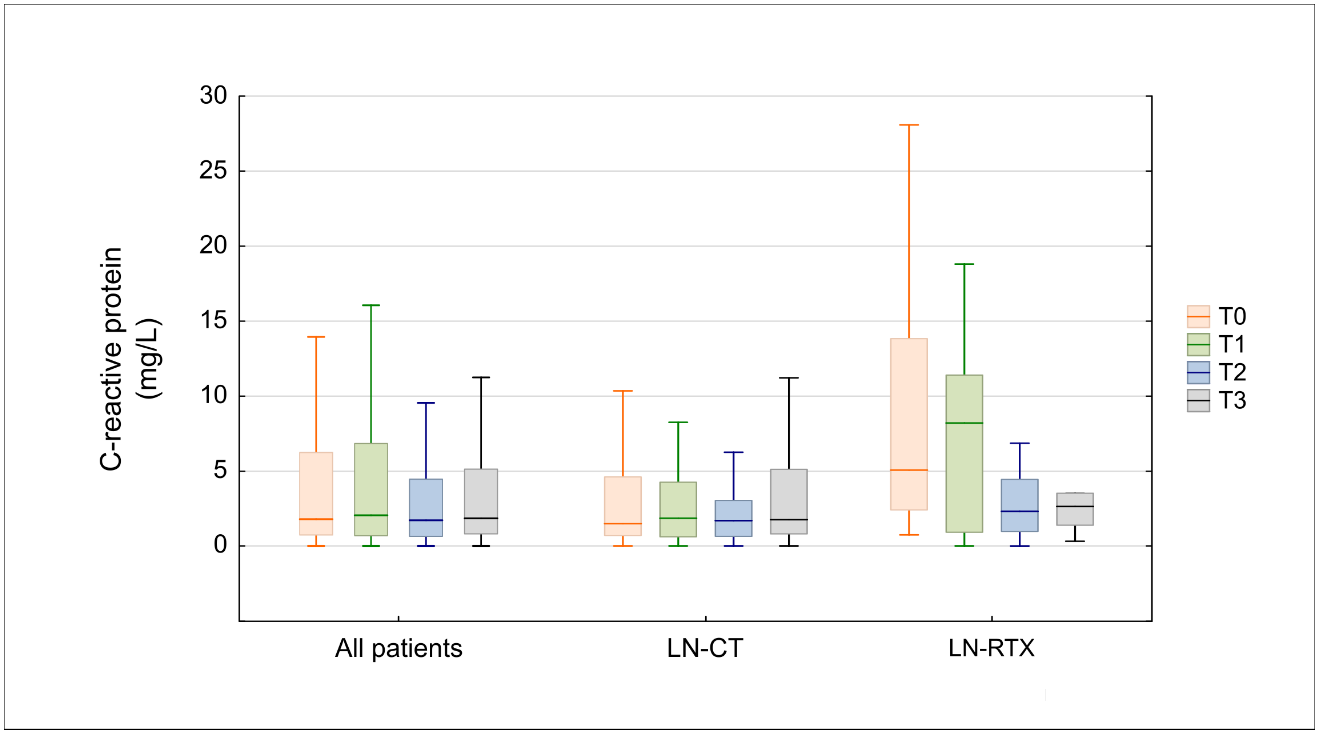

Figure S1. Longitudinal changes in C-reactive protein levels in lupus nephritis patients during the COVID-19 pandemic. Box-and-whisker plots illustrating C-reactive protein (CRP) concentrations (mg/L) in all patients with lupus nephritis, as well as in subgroups of conservatively treated patients (LN-CT) and kidney transplant recipients (LN-RTX), assessed at four time points: T0 (pre-pandemic, 2019), T1 (first pandemic year, 2020), T2 (second pandemic year, 2021), and T3 (post-pandemic period, 2023). Plots show median values, interquartile ranges (IQR), and complete data distribution (minimum–maximum).

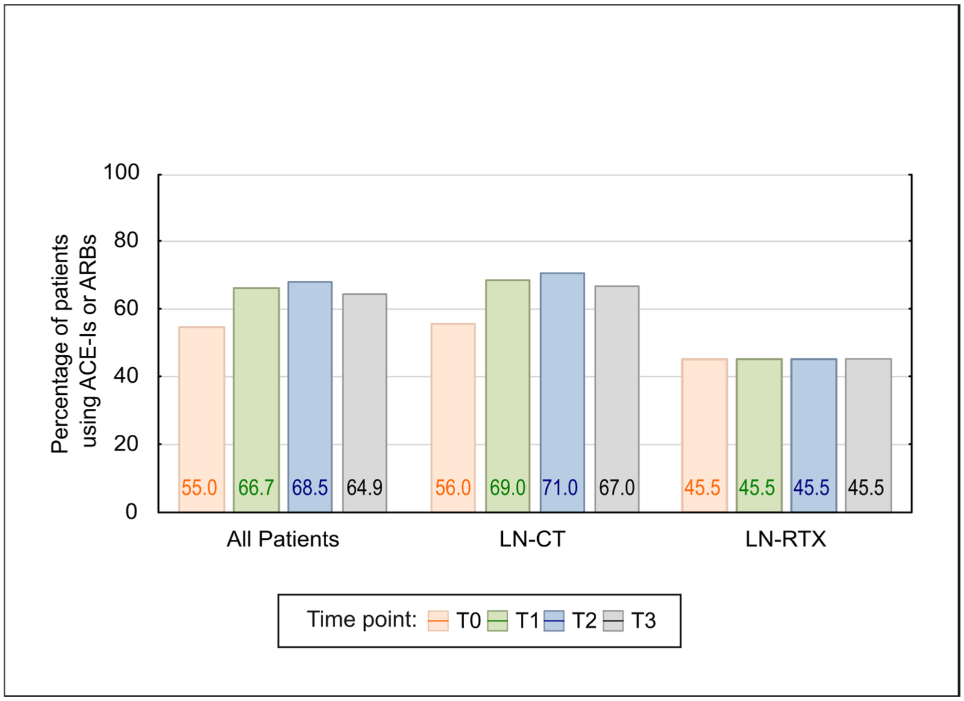

Figure S2. Trends in ACE-Is/ARBs use over the COVID-19 pandemic in patients with lupus nephritis.
Bar chart illustrating the proportion of patients with lupus nephritis receiving angiotensin-converting enzyme inhibitors (ACE-Is) or angiotensin receptor blockers (ARBs) at four time points: T0 (pre-pandemic, 2019), T1 (first pandemic year, 2020), T2 (second pandemic year, 2021), and T3 (post-pandemic period, 2023). Data are shown for the overall cohort ("All Patients") and for treatment modalities: conservatively treated (LN-CT) and kidney transplant recipients (LN-RTX). A progressive increase in ACE-I/ARB use was observed in the LN-CT group, while utilization remained unchanged in the LN-RTX group across all time points.

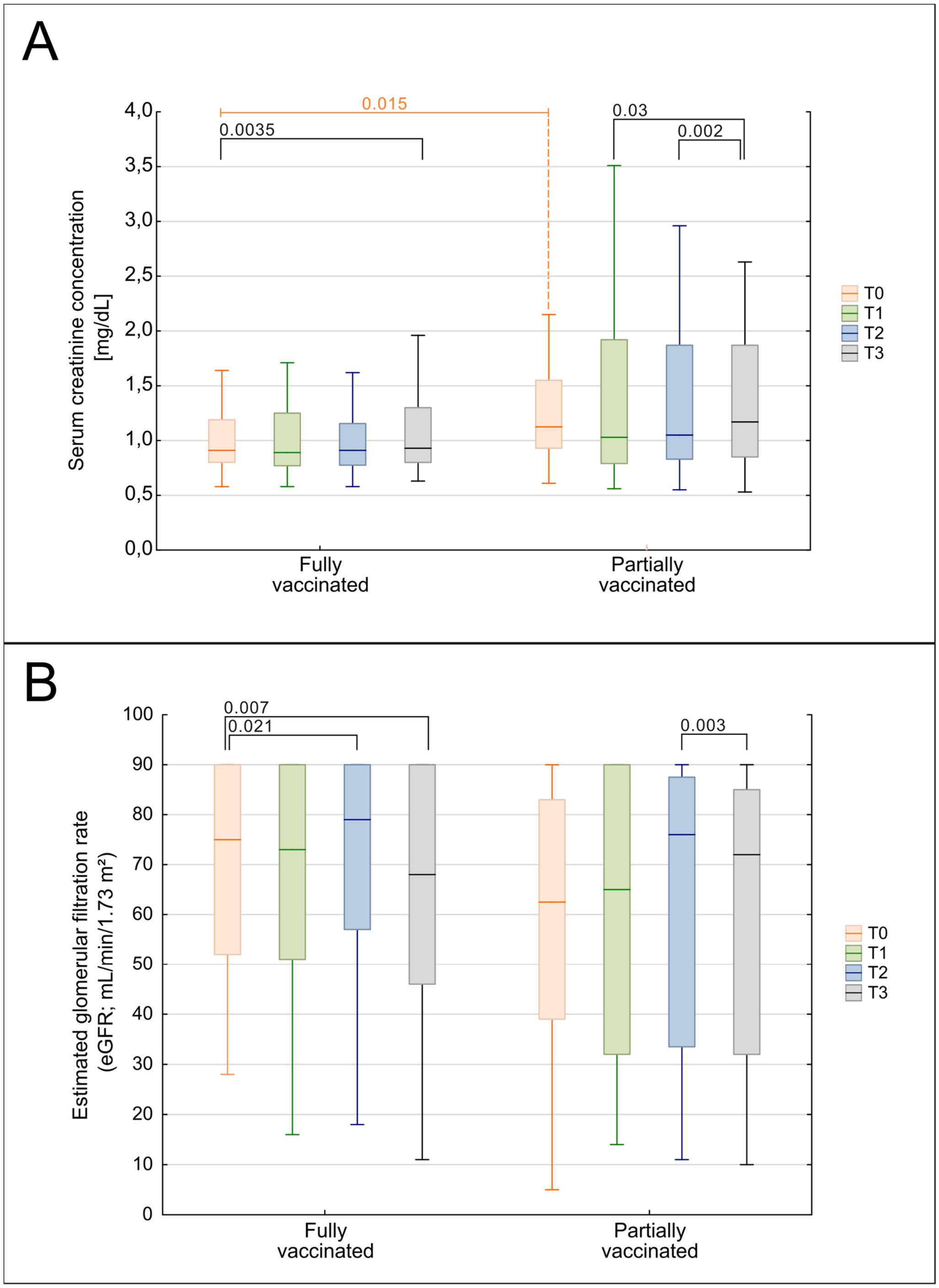

Figure S3. Comparison of renal function over time in fully vaccinated and partially vaccinated patients.
(A) Serum creatinine concentration (sCr, mg/dL) and (B) estimated glomerular filtration rate (eGFR, mL/min/1.73 m²; CKD-EPI) assessed at four time points: T0 – the year preceding the COVID-19 pandemic (2019); T1 – the first pandemic year (2020); T2 – the second pandemic year (2021); and T3 – the post-pandemic period (2023). Patients were stratified into two groups based on COVID-19 vaccination completeness: fully vaccinated and partially vaccinated. Box-and-whisker plots display medians, 25th–75th percentiles, and non-outlier ranges.
P-values shown on the plots denote within-group pairwise comparisons between time points, calculated using the Wilcoxon signed-rank test. Between-group comparisons (fully vs partially vaccinated), where performed, used the Mann–Whitney U test with continuity correction but are not displayed. Only statistically significant results ($p < 0.05$) are shown

Table S1. Peripheral blood parameters over time.

Parameter	Time point	LN-CT Median (IQR)	LN-RTX Median (IQR)	*p*-Value (between groups)
Hemoglobin (g/dL)	T0	13.1 (12.1–14.3)	12.6 (11.2–13.2)	0.272
	T1	12.8 (11.9–13.9)	12.5 (11.0–13.4)	0.459
	T2	13.0 (12.0–14.0)	11.9 (11.0–13.5)	0.456
	T3	13.2 (12.0–14.1)	13.0 (12.0–13.6)	0.633
White blood cells (×10⁹/L)	T0	6.3 (5.1–8.3)	8.6 (6.5–10.4)	0.112
	T1	6.9 (5.2–8.6)	8.3 (6.3–10.5)	0.213
	T2	6.8 (5.0–8.2)	7.4 (5.5–9.4)	0.346
	T3	6.9 (5.0–8.5)	8.7 (6.3–10.6)	0.273
Neutrophils (×10⁹/L)	T0	4.2 (3.1–5.6)	5.5 (4.1–7.1)	0.594
	T1	4.4 (3.0–5.9)	5.2 (4.0–6.5)	0.248
	T2	4.3 (3.2–5.5)	4.3 (3.2–6.0)	0.173
	T3	4.5 (3.0–5.7)	5.6 (4.1–7.3)	0.767
Lymphocytes (×10⁹/L)	T0	1.7 (1.5–2.1)	1.7 (1.4–1.9)	0.958
	T1	1.8 (1.5–2.2)	1.8 (1.5–2.1)	0.051
	T2	1.8 (1.5–2.1)	2.2 (1.8–2.5)	0.213
	T3	1.7 (1.5–2.2)	2.2 (1.8–2.5)	0.323
Platelets (×10⁹/L)	T0	236 (190–292)	216 (187–286)	0.289
	T1	239 (200–285)	234 (172–283)	0.331
	T2	235 (190–290)	236 (200–287)	0.246
	T3	235 (185–288)	238 (184–287)	0.521

Values are presented as medians with interquartile ranges (IQR) due to non-normal distribution, as assessed by the Shapiro–Wilk test. Laboratory findings were analyzed at four distinct time points: T0 – the period preceding the COVID-19 pandemic (2019); T1 – the first pandemic year (2020); T2 – the second pandemic year (2021); and T3 – the post-pandemic period (2023).

P-values denote between-group comparisons at each time point (Mann–Whitney U-test). A two-tailed $p < 0.05$ was considered statistically significant.

Table S2. Immunosuppressive therapy over time.

Parameter	Time point	LN-CT	LN-RTX	*p*-Value (between groups)
Glucocorticoid use (%)	T0	95.0	100.0	NS
	T1	93.0	100.0	NS
	T2	94.0	100.0	NS
	T3	93.0	100.0	NS
Immunosuppressives use (%)	T0	60.0	100.0	0.007
	T1	73.0	100.0	NS
	T2	72.0	100.0	NS
	T3	71.0	100.0	NS
Mycophenolate mofetil use (%)	T0	48.0	81.8	NS
	T1	57.0	72.7	NS
	T2	61.0	72.7	NS
	T3	59.0	72.7	NS
Azathioprine use (%)	T0	1.0	0.0	NS
	T1	1.0	0.0	NS
	T2	1.0	0.0	NS
	T3	3.0	0.0	NS
Cyclosporine A use (%)	T0	11.0	9.09	NS
	T1	13.0	9.09	NS
	T2	15.0	9.09	NS
	T3	18.0	9.09	NS
Tacrolimus use (%)	T0	2.0	72.73	< 0.001
	T1	3.0	72.73	< 0.001
	T2	3.0	72.73	< 0.001
	T3	3.0	72.73	< 0.001
Cyclophosphamide use (%)	T0	3.0	0.0	NS
	T1	4.0	0.0	NS
	T2	2.0	0.0	NS
	T3	0.0	0.0	NS
Antimalarial Drugs use (%)	T0	37.0	9.1	NS
	T1	48.0	9.1	0.0275
	T2	53.0	0.0	0.0023
	T3	55.0	9.1	0.0105

Values are presented as medians with interquartile ranges (IQR) or percentages, depending on variable type. Therapeutic regimens were analyzed at four time points: T0 – the year preceding the COVID-19 pandemic (2019); T1 – the first pandemic year (2020); T2 – the second pandemic year (2021); and T3 – the post-pandemic period (2023). *P*-values denote between-group comparisons at each time point (Mann–Whitney U test for continuous variables and Fisher's exact test for categorical variables). Only statistically significant *p*-values ($p < 0.05$) are reported; all non-significant comparisons are marked as NS (non-significant). A two-tailed *p*-value < 0.05 was considered statistically significant.

Table S3. Lupus Nephritis Activity over time. Longitudinal changes in lupus nephritis activity and renal parameters in the LN-CT and LN-RTX groups across study time points (T0–T3).

**Parameter**	**Time point**	**LN-CT**	**LN-RTX**	***p*-Value** (between groups)
Active nephropathy (%)	T0	56%	36,4%	0,34
	T1	48,7%	45,5%	1,00
	T2	40,8%	18,2%	0,19
	T3	49%	36,4%	0,53
Proteinuria (%)	T0	38,0%	27,3%	0,74
	T1	41,0%	27,3%	0,52
	T2	39,5%	18,2%	0,32
	T3	33,0%	18,2%	0,49
Erytrocyturia score (mean ± SD)	T0	0,60 ± 0,99	0,50 ± 0,71	0,90
	T1	0,34 ± 0,66	0,11 ± 0,33	0,46
	T2	0,11 ± 0,32	0,22 ± 0,44	0,60
	T3	0,44 ± 0,81	0,43 ± 1,13	0,63
Erytrocyturia (%)	T0	33,0%	36,4%	1,00
	T1	32,1%	18,2%	0,49
	T2	42,9%	18,2%	0,19
	T3	43,5%	18,2%	0,18

Data are presented as percentages for categorical variables and as means ± standard deviations (SD) for the erythrocyturia score. Between-group comparisons (LN-CT vs. LN-RTX) at each time point were performed using the Mann–Whitney U test for continuous variables and Fisher's exact test for categorical variables. Longitudinal changes within groups were assessed using Friedman ANOVA with Kendall's W as a measure of concordance. Bold p-values indicate statistical significance ($p < 0.05$).

Table S4. Adjusted linear mixed-effects models for longitudinal changes in serum creatinine and eGFR (T0–T3)

Predictor	β (sCr, mg/dL)	95% CI	p-Value	β (eGFR, mL/min/1.73 m²)	95% CI	p-Value
**Time (per time point)**	+0.108	-0.004 to +0.221	0.060	–1.67	–3.15 to –0.19	**0.027**
Vaccination (Yes vs No)	+0.34	–0.01 to +0.69	0.058	–8.8	–18.7 to +1.1	0.081
Time × Vaccination	–0.095	–0.230 to +0.039	0.160	+0.37	–1.39 to +2.12	0.680
Age (per year)	+0.004	–0.003 to +0.010	0.290	–0.21	–0.42 to +0.01	0.062
Male sex	+0.06	–0.10 to +0.23	0.450	–1.9	–5.7 to +1.9	0.330
Hypertension	+0.03	–0.12 to +0.18	0.660	–1.1	–4.4 to +2.1	0.490
Diabetes	+0.05	–0.13 to +0.24	0.580	–0.8	–4.9 to +3.2	0.680
Immunosuppression	+0.09	–0.11 to +0.28	0.380	–1.6	–5.6 to +2.4	0.430
**Active nephropathy**	+0.27	0.08 to 0.45	**0.005**	–3.95	–6.64 to –1.26	**0.004**
GCS dose (per mg prednisone/day)	+0.004	–0.001 to +0.009	0.110	–0.17	–0.35 to +0.00	0.050

Linear mixed-effects models were fitted with a random intercept for each participant (ID) to account for repeated measures across four time points (T0–T3). Time was coded as an ordinal variable (0 = T0, 1 = T1, 2 = T2, 3 = T3). Vaccination (Yes/No) was defined as receipt of at least one dose of a COVID-19 vaccine before the corresponding time point. Active nephropathy was determined based on clinical and laboratory assessment at each visit. Daily glucocorticoid dose was analyzed as a continuous prednisone-equivalent measure. Immunosuppression denotes the use of non-glucocorticoid immunosuppressive therapy.

β coefficients represent adjusted mean differences in serum creatinine (mg/dL) or eGFR (mL/min/1.73 m²) associated with each predictor. The coefficient for **Vaccination** reflects overall cross-sectional differences in renal function between vaccinated and unvaccinated patients. In contrast, the **Time × Vaccination** interaction assesses whether vaccination modifies the rate of change in renal function **over time**. A two-sided $p < 0.05$ was considered statistically significant.

Statistically significant results are shown in **bold**.

Table S5. Summary of Major Longitudinal Changes in Clinical Parameters and Treatments (T0–T3)

Parameter	T0 (Baseline)	T3 (Post-pandemic)	Δ (T3–T0)	*P*-value (longitudinal)	Main Finding
eGFR (mL/min/1.73 m²)	70.1 ± 21,7	68.0 ± 24,2	−2.1	0.0025	Small but significant decline driven by LN-CT
Serum creatinine (mg/dL)	1.19 ± 1,17	1.24 ± 0,97	+0.05	0.0018	Significant increase; LN-RTX stable
Active nephropathy (%)	54.3%	33.3%	−21.0%	0.0398	Weak but significant decline; small effect size
Proteinuria (%)	36.7%	33.3%	−3.4%	NS	Stable; no clinically relevant change
Erythrocyturia score (0–3)	0.52 ± 0.88	0.30 ± 0.61	−0.22	0.003	Significant decrease in LN-CT only
GCs daily dose (mg/day)	8.62 ± 8,10	5,92 ± 3,17	−2.5	<0.001	Significant tapering at T2 and T3
MMF dose (mg/day)	850 ± 393,9	700 ± 248,5	−250	0.0231	Significant reduction in LN-CT; stable in LN-RTX
ACEIs/ARBs use (%)	55.0%	64.9%	+9.9%	NS	Non-significant increase
Vitamin D supplementation (%)	53.2%	81.1%	+27.9%	<0.001	Marked increase during the pandemic

Data are presented as means ± standard deviations (SD) for continuous variables and percentages (%) for categorical variables. Δ denotes the absolute change between baseline (T0) and the post-pandemic assessment (T3). *P-values* refer to longitudinal analyses performed using Friedman ANOVA (for continuous non-normally distributed variables), Kendall's W as a measure of concordance, or Wilcoxon signed-rank tests for post-hoc comparisons, as appropriate. For categorical variables, repeated-measures changes were assessed using Cochran's Q test. All numerical values (T0, T3, and Δ) refer to the overall cohort. The study population consisted of two predefined groups – patients with preserved native kidney function managed conservatively (LN-CT) and kidney transplant recipients with a history of lupus nephritis (LN-RTX). Interpretations specific to these subgroups, where provided in the 'Main Finding' column, are derived from stratified analyses detailed in the main text.

Abbreviations: eGFR, estimated glomerular filtration rate; sCr, serum creatinine; GCs, glucocorticoids; MMF, mycophenolate mofetil; ACEIs, angiotensin-converting enzyme inhibitors; ARBs, angiotensin receptor blockers; SD, standard deviation.
